# Risk factors for esophageal cancer in a low-incidence area of Brazil

**DOI:** 10.1590/S1516-31802013000100005

**Published:** 2013-02-01

**Authors:** Orlando Milhomem Mota, Maria Paula Curado, José Carlos Oliveira, Edesio Martins, Daniela Medeiros Milhomem Cardoso

**Affiliations:** I MD. Head of Digestive Surgery Service, Hospital Araújo Jorge, Associação de Combate ao Câncer de Goiás (ACCG), and Postgraduate Program on Health Sciences, School of Medicine, Universidade Federal de Goiás (UFG), Goiânia, Goiás, Brazil.; II MD, PhD. Senior Researcher, International Prevention Research Institute and Population-Based Cancer Registry of Goiânia, Associação de Combate ao Câncer de Goiás (ACCG), Goiânia, Goiás, Brazil.; III MD, PhD. Head of Head and Neck Surgery Service, Hospital Araújo Jorge, and Population-Based Cancer Registry of Goiânia, Associação de Combate ao Câncer de Goiás (ACCG), Goiânia, Goiás, Brazil.; IV MHSc. Epidemiologist and doctoral student, Postgraduate Program on Health Sciences, School of Medicine, Universidade Federal de Goiás (UFG), and Population-Based Cancer Registry of Goiânia, Associação de Combate ao Câncer de Goiás (ACCG), Goiânia, Goiás, Brazil.; V MD. Endoscopist, Digestive Surgery Service, Hospital Araújo Jorge, Associação de Combate ao Câncer de Goiás (ACCG), and master’s degree student, Postgraduate Program on Health Sciences, School of Medicine, Universidade Federal de Goiás (UFG), Goiânia, Goiás, Brazil.

**Keywords:** Esophagus, Esophageal neoplasms, Epidemiology, Risk factors, Food habits, Esôfago, Neoplasias esofágicas, Epidemiologia, Fatores de risco, Hábitos alimentares

## Abstract

**CONTEXT AND OBJECTIVES:**

Esophageal cancer is the eighth commonest type of cancer worldwide, occupying sixth place in terms of mortality. Smoking and alcohol use are known risk factors for this type of cancer. The aim here was to evaluate the risk factors for esophageal cancer in a low-incidence area.

**DESIGN AND SETTING:**

Case-control study in Goiânia, with 99 cases of esophageal cancer and 223 controls.

**METHODS:**

The variables were sociodemographic, dietary, occupational and lifestyle data. The sample was analyzed using the chi-square test, Mann-Whitney test and Mantel-Haenszel approach for multivariate analysis. Odds ratios (OR) were calculated with 5% significance and 95% confidence intervals.

**RESULTS:**

The risk of esophageal cancer was higher in patients ≥ 55 years (OR = 1.95; P < 0.001). Patients from rural areas were at greater risk of esophageal cancer (OR = 4.9; P < 0.001). Smoking was a risk factor among the cases (OR = 3.8; P < 0.001), as was exposure to woodstoves (OR = 4.42; P < 0.001). The practice of oral sex was not a risk factor (OR = 0.45; P = 0.04). Consumption of apples, pears, vegetables, cruciferous vegetables and fruit juices were protective against esophageal cancer.

**CONCLUSION:**

In a region in which the incidence of esophageal cancer is low, the most significant risk factors were exposure to woodstoves, smoking and living in rural areas.

## RESUMO

**CONTEXTO E OBJETIVOS:**

O câncer de esôfago é o oitavo tipo mais comum em todo o mundo, ocupando o sexto lugar em termos de mortalidade. Fatores de risco conhecidos para esse tipo de câncer são o consumo de tabaco e o álcool. O objetivo foi avaliar os fatores de risco para câncer de esôfago em uma área de baixa incidência.

**TIPO DE ESTUDO E LOCAL:**

Estudo caso-controle em Goiânia, com 99 casos de câncer de esôfago e 223 controles.

**MÉTODOS:**

As variáveis foram dados sociodemográficos, alimentares, ocupacionais e de estilo de vida. A amostra foi analisada pelo teste do qui-quadrado, Mann-Whitney e de Mantel-Haenszel para análise multivariada. Foram calculados a *odds ratio* (OR) com significância em 5% e o intervalo de confiança de 95%.

**RESULTADOS:**

O risco de câncer de esôfago foi maior em pacientes ≥ 55 anos (OR = 1,95, P < 0,001). Pacientes de áreas rurais estavam em risco maior de câncer de esôfago (OR = 4,9; P < 0,001). O tabagismo foi um fator de risco entre os casos (OR = 3,8; P < 0,001), bem como exposição ao fogão a lenha (OR = 4,42; P < 0,001). A prática do sexo oral não foi fator de risco (OR = 0.45; P = 0,04). Consumo de maçãs, peras, legumes, vegetais crucíferos e sucos de frutas foi protetor contra o câncer de esôfago.

**CONCLUSÃO:**

Em uma região em que a incidência de câncer de esôfago é baixa, os fatores de risco mais significativos foram a exposição a fogão a lenha, tabagismo e viver em zona rural.

## INTRODUCTION

Esophageal cancer is the eighth most common type of cancer worldwide, occupying sixth place in terms of mortality. Projections for 2015 indicate that approximately 579,554 new cases and 489,123 deaths will occur from this type of cancer in both genders worldwide.^1^ Esophageal cancer is the third most common malignancy of the digestive tract after stomach and colorectal cancer.^2^ The worldwide distribution of esophageal cancer is heterogenous, with low rates in industrialized and developed countries except for Japan.^3^ In countries in which the incidence is high, such as China (73.2 per 100,000)^4^ and Japan, cases are diagnosed from the age of 30 years onwards, and the incidence increases with age. In Latin America, the incidence and mortality rates are low in Mexico and Peru; however, in Brazil, Argentina, Chile, Uruguay and Puerto Rico, these rates are found to be higher.^5^

In Brazil, the incidence ranges from 1 to 18 per 100,000 inhabitants and is higher in the southern part of the country (9 to 18 per 100,000), intermediate in the central-western and northeastern regions (4 to 9 per 100,000) and low in the north (1 to 2 per 100,000).^6^ Like in eastern Asian countries, cases start to be diagnosed from 30 years of age onwards.^7^

Risk factors such as alcohol, smoking, fungal toxins, nutritional deficiencies, foods, hot liquids, chemical carcinogens, occupational exposure and infectious agents are involved in the genesis of esophageal tumors. The human papillomavirus (HPV) has a role in the genesis of these tumors and the most common HPV genotypes involved are HPV 16, 18 and 59.^5^

Smoking is one of the main risk factors for squamous cell carcinoma of the esophagus, particularly if black tobacco is used. When consumption exceeds 50 packs/year, the risk becomes more than 40 times greater than among non-smokers.^8^ Therefore, smoking is an independent risk factor. Furthermore, when smoking is associated with alcohol consumption, the effect is synergic.^7^^,^^8^^,^^9^^,^^10^

According to Grønbaek et al.,^9^ alcohol intake exceeding 80 grams per day increases the risk of esophageal cancer to more than 100 times the risk among non-drinkers. Fermented drinks (beer and wine) consumed in moderation have been found to be a low risk factor.^9^^,^^11^^,^^12^

Regarding dietary habits, intake of over 145 grams/day of red meat or processed meat products constitutes a risk factor for esophageal cancer, according to studies conducted in Uruguay,^13^ Italy,^14^ Canada^15^ and the United States.^16^

However, consumption of fruit and raw vegetables rich in vitamins A, C and E, minerals such as selenium, molybdenum and zinc,^11^ folates, flavonoids and fiber^12^ plays a protective role against esophageal cancer, through functioning as an endogenous blocker of nitrogenated compounds. Therefore, lifestyle associated with dietary habits constitutes a determining factor in the genesis of malignant tumors of the esophagus.^11^

Studies on the risk factors for esophageal cancer have been conducted in areas in which the incidence of this disease is high. In Brazil, the incidence is intermediate, but there are significant regional variations. The incidence in Goiânia, state of Goiás, was estimated at 7.60 per 100,000 men and 1.98 per 100,000 women in 2008.^7^

Few reports have been published in the literature on the risk factors in low-risk populations. Nevertheless, the importance of better understanding of the risk factors for esophageal cancer has been highlighted, especially because of the high lethality of this tumor. The possibility of identifying unknown risk factors, in addition to those already known, could provide further information for prevention of this type of tumor.

## OBJECTIVE

To analyze the risk factors for esophageal cancer in a low-incidence region of Brazil.

## METHODS

This study was part of a multicenter study organized by the International Agency for Research on Cancer (IARC), which has its headquarters in Lyon, France. It was a hospital-based, case-control study that began in August 1998 and terminated in June 2003.

### Sample size

By accepting alpha of 0.05 with power of 0.80 and an estimated prevalence of exposure risk among controls of 10%, it was estimated that a sample of 99 cases and 223 controls would be required in order to detect an odds ratio (OR) of 1.8.

### Inclusion criteria for cases and controls

Patients with squamous cell carcinoma of the esophagus (ICD-O3 C15.0-C15.9), receiving care at the Department of Digestive Tract Diseases of Hospital Araújo Jorge, Anti-Cancer Association of Goiás (Associação de Combate ao Câncer em Goiás, ACCG) were enrolled in the study as cases. Patients selected at two public hospitals not specializing in oncology in Goiânia, the Goiânia Emergency Hospital (Hospital de Urgências de Goiânia, HUGO) and the Goiânia General Hospital (Hospital Geral de Goiânia, HGG), were admitted as controls. All the control patients had been living in Goiás for at least one year prior to the study and had no history of esophageal cancer. The controls were matched to the cases for gender, age (± five years) and place of residence (urban or rural), in order to minimize any bias in selecting the study population. Patients with difficulty in communicating and in responding to questionnaires or signing the informed consent form were excluded from the study.

After receiving an explanation regarding the objectives of the study and reading the informed consent form, the cases and controls who agreed to participate in the study signed the informed consent form and were interviewed in accordance with the standardized questionnaire.

## RESULTS

A total of 99 cases of esophageal carcinoma were identified and 223 controls were enrolled, in proportions of two controls for every case. In the case group, 77 patients (77.8%) were male and 22 (22.2%) were female, while in the control group 181 (81.3%) were male and 42 (18.7%) were female. The majority of the cases were over 55 years of age (n = 68; 68.7%) and likewise the controls (n = 118, 52.9%) (OR 1.95; 95% confidence interval, CI: 1.8-3.21; P = 0.008). With regard to marital status, 94% of the cases and 77% of the controls were married. Thirty cases lived in urban areas, while 69 were from rural regions (Table 1).

**Table 1.** Numbers and percentages of cases of esophageal cancer and controls according to selected sociodemographic variables. Goiânia, 1998 to 2003

Variable	Case		Control		P-value^*^	OR (95% CI)
	n	%	n	%		
Gender						
Male	77	77.8	182	81.3		1
Female	22	22.2	42	18.7	0.482	0.86 (0.48-1.53)
Age						
³ 55 years	68	68.7	118	52.9	0.008	1.95 (1.18-3.21)
< 55 years	31	31.3	105	47.1		1
Marital status						
Married	93	93.9	147	77.0	< 0.001	4.64 (1.90-11.31)
Single	6	6.1	44	23.0		1
Residence						
Urban	30	30.3	152	68.2		1
Rural	69	69.7	71	31.8	< 0.001	4.92 (2.94-8.22)
Education level^†^						
Illiterate	30	30.3	53	23.8		1
1^st^-4^th^ grade	59	59.6	153	68.6	0.143	0.68 (0.39-1.16)
5^th^-8^th^ grade	10	10.1	15	6.7	0.639	1.17 (0.47-2.94)
High school	0	0.0	2	0.9	0.286	0.69 (0.61-0.79)

^*^Chi-square test; ^†^There were no subjects with university education among either the cases or the controls; OR = odds ratio; CI = confidence interval.

In the control group, 78 patients (35%) had been hospitalized due to external causes, while 55 (24.7%) had circulatory problems (Table 2).

No statistically significant differences were found between the cases and controls with regard to gender or education level.

**Table 2.** Numbers and percentages of controls according to diagnosis

Diagnosis	n	%
Infectious and parasitic diseases	8	3.6
Blood-related diseases	5	2.2
Endocrine diseases	8	3.6
Diseases of the nervous system	6	2.7
Diseases of the circulatory system	55	24.7
Diseases of the respiratory system	17	7.6
Skin and subcutaneous diseases	5	2.2
Diseases of the skeletal system and connective tissues	18	8.1
Diseases of the genital/urinary tract	14	6.3
External causes of injury	78	35.0
External causes of accidental trauma	9	4.0
Total	223	100.0

The risk of esophageal cancer was greater among patients over 55 years of age (OR = 1.95; 95% CI: 1.18-3.20; P = 0.01) and among married patients (OR = 4.63; 95% CI: 1.92-11.3; P < 0.001). The risk was higher among individuals living in rural areas than among those living in urban areas (OR = 4.9; 95% CI: 2.9-8.2; P < 0.001) (Table 1).

Smoking was more common among the cases (OR = 3.87; 95% CI: 1.90-7.89; P < 0.001), as was alcohol consumption. The practice of oral sex did not constitute a risk factor for esophageal cancer (OR = 0.45; 95% CI: 0.21-0.98; P = 0.04) (Table 3).

**Table 3.** Numbers and percentages of patients, according to lifestyle. Goiânia, 1998 to 2003

Variable	Case		Control		P-value^*^	OR (95% CI)
	n	%	n	%		
Smoking						
No	12	12.1	54	24.2		1
Yes	62	62.6	72	32.3	< 0.001	3.87 (1.90-7.89)
Previously	25	25.3	97	43.5	0.70	1.02 (0.89-1.19)
Alcohol intake						
No	15	15.2	48	21.5		1
Yes	44	44.4	94	42.2	0.24	1.49 (0.76-2.96)
Previously	40	40.4	81	36.3	0.19	1.14 (0.94-1.37)
Yerba mate						
No	93	93.9	205	91.9		1
Yes	2	2.0	7	3.1	0.56	1.13 (0.79-1.61)
Previously	4	4.0	11	4.9	0.71	1.25 (0.38-4.02)
Oral sex						
No	90	90.9	182	82.0		1
Yes	9	9.1	40	18.0	0.04	0.45 (0.21- 0.98)
Use of woodstove						
No	66	66.7	168	89.8		1
Yes	33	33.3	19	10.2	< 0.001	4.42 (2.354-8.32)
Woodstove was used in childhood home						
No	10	10.1	17	10.2		1
Yes	89	89.9	150	89.8	0.98	1.01 (0.44-2.29)

^*^Chi-square test.

Use of a woodstove for cooking was also found to be a significant risk factor for esophageal cancer (OR = 4.42; 95% CI: 2.3-8.31; P < 0.001) (Table 3).

**Figure 1.**
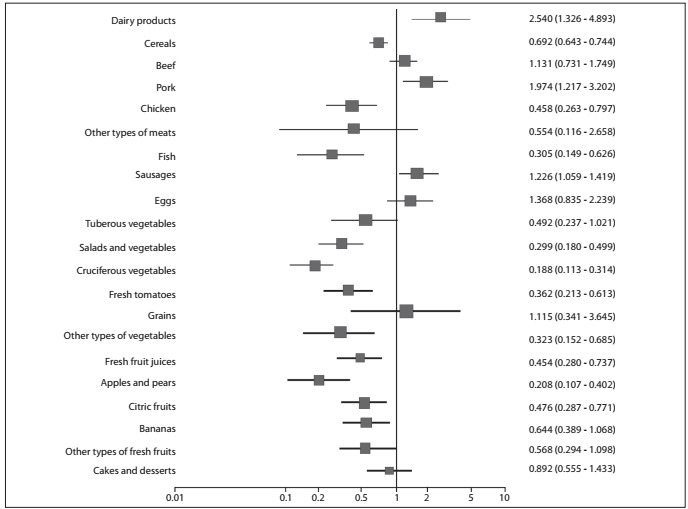
Univariate analyses on the dietary habits of the cases and controls with regard to esophageal cancer (chi-square test and odds ratio).

In the univariate analysis, consumption of raw vegetables and salads was found to be a protective factor, as was the consumption of fresh fruit juice, apples and pears, citric fruits, bananas, fish and chicken. Also in the univariate analysis, consumption of pork meat, dairy products and processed meats was found to be positively and significantly associated with esophageal cancer (Figure 1).

**Figure 2.**
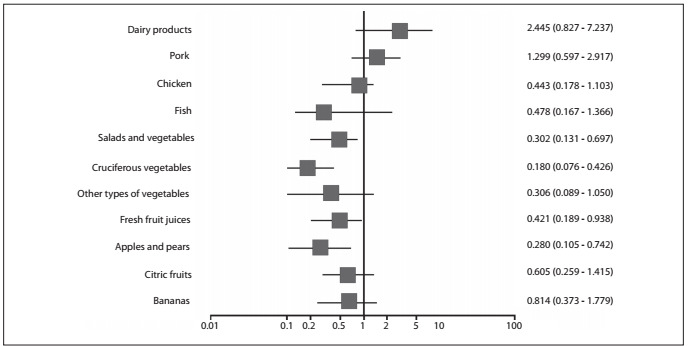
Multivariate analyses on the dietary habits of the cases and controls with regard to esophageal cancer (Mantel-Haenszel test).

Multivariate analysis, adjusted for confounding factors such as smoking, alcohol intake and urban or rural residence, confirmed that consumption of apples, pears, salads and vegetables, fruit juice and cruciferous vegetables constituted a protective factor against esophageal cancer (Figure 2).

## DISCUSSION

The incidence of esophageal cancer varies widely even within the same country, due to different types of exposure and the genetic susceptibility of individuals to different lifestyles.^17^ The incidence rates of esophageal cancer in Brazil are heterogenous, and cases are generally diagnosed at advanced stages. At such times, treatment is a challenge for oncologists.

Goiânia is the second largest city in central-western Brazil, in the state of Goiás, with 1,301,892 inhabitants: 1,296,969 in urban regions and 4,923 in rural regions. The life expectancy in Goiânia was about 71.3 years for both genders in 2003.^18^

In the present study, patients over 55 years of age were found to have higher risk of esophageal cancer. In high-incidence areas such as China, Japan and Iran, cases of this disease start to be diagnosed from the age of 30 years onwards, reaching a peak at 55 years of age.^1^^,^^3^^,^^19^^,^^20^^,^^21^ The higher risk of esophageal cancer from 55 years of age onwards is similar to that found for the majority of solid tumors and tumors of the digestive tract, in which the incidence increases with age due to the latency period and the duration of exposure to risk factors.

Living in a rural area was found to be a risk factor for esophageal cancer in this study. Other studies in high-incidence regions have also shown that patients living in rural areas are more likely to develop esophageal cancer.^21^^,^^22^^,^^23^^,^^24^ Although Goiânia is a low-incidence region in Brazil, it was found that the patients from rural areas were at high risk of esophageal cancer.

In China, the habit of cooking on a woodstove inside the home is very common, thereby exposing individuals to the effects of soot inhalation.^7^^,^^9^^,^^10^ This has been shown to be a risk factor for esophageal cancer. In Brazil, the habit of cooking food on a woodstove inside the home is common, not only in rural areas but also in urban regions of the country.^18^ In Goiás, the practice is common among rural populations and was identified as a statistically significant single risk factor in the present study. Dispersal of soot from burning wood (smoke) and the consequent exposure to it may represent risk factors for carcinoma of the esophagus.^22^ Further studies should be carried out to evaluate the use of woodstoves and the duration of exposure to soot and smoke in order to establish whether this exposure can be considered to be a definitive risk factor for esophageal cancer.^25^ We identified that cooking on a woodstove, which is a common habit in central parts of Brazil, was a risk factor.

In the present study, smoking was found to be a risk factor for esophageal cancer, increasing the likelihood of developing the disease by a factor of four. This result is similar to what was reported by Lee et al.^26^ Following combustion, tobacco is known to dissociate into more than 4,720 compounds, and more than sixty of them are considered to be carcinogenic. Tar is one of the principal components and contains benzopyrene and aromatic amines, of which nitrosamine has the highest level of carcinogenicity.^27^^,^^28^^,^^29^^,^^30^^,^^31^

Alcohol intake was considered to be a strong risk factor for esophageal cancer in the studies conducted by Vioque et al.,^7^ Schütze et al.^10^ and Dietz et al.^22^ Lee et al.^26^ reported that the risk was up to 14 times higher when the intake exceeded 900 g/day/year. In Goiás, the overall prevalence of alcohol consumption in 2008 was 17.6% (28% among males and 7% among females). The highest prevalence of alcohol consumption in Brazil is found in Belém (37.2%) and the lowest in Curitiba (4.6%).^32^ Therefore, this intermediate prevalence of alcohol consumption in Goiás may explain the low risk of esophageal cancer found in the present study. In the multivariate analysis, no synergic effect was found between smoking and alcohol consumption as a risk factor for esophageal cancer.^33^^,^^34^^,^^35^^,^^36^

Consumption of yerba mate is not common in Goiás, and only ten individuals in the case group (4%) reported this habit. It was therefore not possible to confirm that this habit is a risk factor for esophageal cancer. According to Dietz et al.,^22^ consumption of yerba mate in the form of a hot infusion (referred to in Brazil as *chimarrão*) constitutes a risk factor (OR = 3.58) and is considered to be a single risk factor when consumption exceeds 1,000 ml/day.^35^^,^^36^ In studies conducted using guinea pigs, Kruel et al.^37^ failed to show any carcinogenic effect from pure, unindustrialized yerba mate; on the other hand, they confirmed that nitrosamine presented greater effect as a carcinogen when associated with ingestion of hot water.

Oral sex was not found to be a risk factor for esophageal cancer in the present study (OR = 0.45; P = 0.04). This is discordant with reports in the literature, particularly the studies conducted by Herrera-Goepfert et al.^5^ and Zhang et al.^4^ These investigators demonstrated that HPV genotype 16 was present in up to 65% of their cases of esophageal cancer. They suggested that there could be two types of transmission: one of them through sexual behavior.^4^^,^^5^ In our study, information about oral sexual practice was only available in a few cases, and for this reason, our statistical assessment was not significant.

A multicenter case-control study by Launoy et al.^17^ showed the independent protective effect of citric fruits, fresh vegetables, oil plants and fresh fish (OR = 0.63). However, in the same study, high consumption of dairy products, salted and smoked fish was found to be a risk factor (OR = 2.67). A cohort study conducted by the Tokyo National Cancer Center^3^ reported that consumption of fresh fruit, citric fruit and cruciferous vegetables had a protective effect against esophageal cancer, reducing the risk of developing this disease by 11%. In the present study, consumption of apples and pears was found to reduce the risk by 27%. Reports in the literature affirm that these foods have a higher content of micronutrients, including carotenoids (alpha-carotene and beta-carotene), vitamin C (an anti-carcinogenic agent, since it inhibits the formation of nitrosamines, amines and nitrites), vitamin E (an antioxidant vitamin), selenium, fiber, lycopene, flavonoids, phenols and proteinase inhibitors, which protect against this type of cancer.^12^^,^^38^^,^^39^^,^^40^ Aune et al.^12^ reported that high consumption of fruit and vegetables (OR = 0.59) was associated with a reduction in the risk of esophageal cancer, and two meta-analyses^41^ have confirmed these findings. These foods exert a protective effect by inhibiting enzymes and reducing oxidative stress and inflammation, and increasing the number of carcinogen-inhibiting agents.^38^^,^^42^

Esophageal cancer is a highly lethal disease and always diagnosed at advanced stages. The present study provides information that can be implemented, both by healthcare professionals and by the population, towards achieving earlier diagnosis, thereby reducing the morbidity and mortality caused by this cancer.

One concern that we have regarding out study is that a case-control design is not the best design for answering questions about risk factors for esophageal cancer. Nonetheless, the present study was able to identify some hypotheses regarding risk factors that may be useful in clinical practice and which might be confirmed through further prospective cohort studies.

Regarding the risk factors for esophageal cancer, many aspects of this topic still require further study in order to achieve better understanding.

## CONCLUSIONS

In the present study, consumption of raw vegetables, citric fruit, apples and pears was confirmed as a protective factor against esophageal cancer. However, the most important risk factors for esophageal cancer in this region of low incidence in Brazil were exposure to woodstoves, smoking, and living in rural areas. Further studies are needed to clarify the mechanisms of exposure to woodstoves and living rurally as risk factors for esophageal cancer.
